# Dose-response relationship between arsenic exposure and the serum enzymes for liver function tests in the individuals exposed to arsenic: a cross sectional study in Bangladesh

**DOI:** 10.1186/1476-069X-10-64

**Published:** 2011-07-08

**Authors:** Khairul Islam, Abedul Haque, Rezaul Karim, Abul Fajol, Ekhtear Hossain, Kazi Abdus Salam, Nurshad Ali, Zahangir Alam Saud, Matiar Rahman, Mashiur Rahman, Rezaul Karim, Papia Sultana, Mostaque Hossain, Anwarul Azim Akhand, Abul Mandal, Hideki Miyataka, Seiichiro Himeno, Khaled Hossain

**Affiliations:** 1 Department of Biochemistry and Molecular Biology, Rajshahi University, Rajshahi-6205, Bangladesh; 2 Department of Applied Nutrition and Food Technology, Islamic University, Kushtia-7003, Bangladesh; 3 FIST, UMP, Gambang 26300, Pahang, Malaysia; 4 Department of Statistics, Rajshahi University, Rajshahi-6205, Bangladesh; 5 Department of Medicine, Bangladesh Institute of Research and Rehabilitation in Diabetes, Endocrine and Metabolic Disorders (BIRDEM), Dhaka, Bangladesh; 6 Department of Genetic Engineering and Biotechnology, Dhaka University, Dhaka-1000, Bangladesh; 7 System Biology Research Center, University of Skövde, P. O. Box 408, SE-541-28 Skövde, Sweden; 8 Laboratory of Molecular Nutrition and Toxicology, Faculty of Pharmaceutical Sciences, Tokushima Bunri University, Tokushima 770-8514, Japan

## Abstract

**Background:**

Chronic arsenic exposure has been shown to cause liver damage. However, serum hepatic enzyme activity as recognized on liver function tests (LFTs) showing a dose-response relationship with arsenic exposure has not yet been clearly documented. The aim of our study was to investigate the dose-response relationship between arsenic exposure and major serum enzyme marker activity associated with LFTs in the population living in arsenic-endemic areas in Bangladesh.

**Methods:**

A total of 200 residents living in arsenic-endemic areas in Bangladesh were selected as study subjects. Arsenic concentrations in the drinking water, hair and nails were measured by Inductively Coupled Plasma Mass Spectroscopy (ICP-MS). The study subjects were stratified into quartile groups as follows, based on concentrations of arsenic in the drinking water, as well as in subjects' hair and nails: lowest, low, medium and high. The serum hepatic enzyme activities of alkaline phosphatase (ALP), aspartate transaminase (AST) and alanine transaminase (ALT) were then assayed.

**Results:**

Arsenic concentrations in the subjects' hair and nails were positively correlated with arsenic levels in the drinking water. As regards the exposure-response relationship with arsenic in the drinking water, the respective activities of ALP, AST and ALT were found to be significantly increased in the high-exposure groups compared to the lowest-exposure groups before and after adjustments were made for different covariates. With internal exposure markers (arsenic in hair and nails), the ALP, AST and ALT activity profiles assumed a similar shape of dose-response relationship, with very few differences seen in the higher groups compared to the lowest group, most likely due to the temporalities of exposure metrics.

**Conclusions:**

The present study demonstrated that arsenic concentrations in the drinking water were strongly correlated with arsenic concentrations in the subjects' hair and nails. Further, this study revealed a novel exposure- and dose- response relationship between arsenic exposure metrics and serum hepatic enzyme activity. Elevated serum hepatic enzyme activities in the higher exposure gradients provided new insights into arsenic-induced liver toxicity that might be helpful for the early prognosis of arsenic-induced liver diseases.

## Background

Arsenic is a ubiquitous element present in food, soil, water and air, and it is released into the environment from both natural and man-made sources [1-4]. Arsenic in drinking water is typically inorganic, and can be present either as As^+3 ^(arsenite) or As^+5 ^(arsenate). In Bangladesh, arsenic in the ground water is primarily in the As^+3 ^form [5,6], which has a higher affinity for thiol groups [6] and is more cytotoxic and genotoxic than As^+5 ^[7,8]. Individuals who accumulate the trivalent intermediates are thought to be greater risk of arsenic-induced diseases [7,8]. The main source of arsenic poisoning is the drinking of arsenic-contaminated ground water. The ingestion of inorganic arsenic is a significant public health hazard in Bangladesh. Bangladesh and West Bengal of India have already experienced the biggest catastrophe in the world due to the presence of the excessive amounts of arsenic in the drinking water [9]. A significant number of cases of toxicity have been reported in different parts of Bangladesh, and tens of millions of additional people are currently at risk of arsenic toxicity throughout the country [10-13]. Arsenic toxicity has been reported to be associated with a variety of cancers, dermatitis, cardiovascular diseases, peripheral neuropathy, diabetes mellitus, renal failure and liver dysfunction [14-20].

The liver is the primary target organ for the metabolism of arsenicals. The major metabolic pathway of inorganic arsenic in humans is its methylation in the liver. The methylation of arsenic has been demonstrated by the presence of monomethylarsonic acid (MMA) and dimethylarsinic acid (DMA) in the urine and bile [21,22]. Monomethylarsonic acid is relatively more genotoxic than the dimethylarsinic acid [23]. Generally the toxicity of arsenic is thought to be largely the result of its reaction with free sulfhydryl groups of enzymes and proteins, followed by cross linkage [24,25]. Oxidative DNA damage, acquired tolerance to apoptosis, enhanced cell proliferation, altered DNA methylation, genomic instability, and aberrant estrogen signaling have been reported to be involved in the liver toxicity caused by arsenic [4,26]. Hepatic cancer and other hepatic disorders are considered to be the major causes of arsenic-related mortality [27-31]. Hepatic function, liver diseases and drug-induced liver injury can be assessed by various routinely ordered liver function tests (LFTs), i.e., clinical investigations that measure the levels of various biomarkers (proteins or enzymes) in the blood. These proteins/enzymes reflect different aspects of a normal functioning liver [32-34]. For example, ALT and AST indicate hepatocellular integrity, and ALP levels indicate whether there is adequate formation of bile and albumin for protein synthesis. Liver function testing is among the most commonly used and primary clinical investigation for the assessment of liver function.

Recently, we reported that arsenic exposure is associated with plasma cholinesterase and LDH activity [35,36]. Both plasma cholinesterase and LDH activities are associated with multi-organ dysfunction including liver intoxication. In a previous study, Guha Mazumder [37] examined a sample of subjects in India and demonstrated that arsenic exposure induced liver enlargement with concomitant elevation in levels of ALP, AST and ALT. At the same time, Nabi and colleagues [38] conducted a study in Bangladesh showing a significant difference in ALP activity in a sample exposed to arsenic compared to that of a non-exposed sample. Another study [39] of arsenic-endemic and non-endemic population in India showed that arsenic elevated the activity of blood proteins/enzymes assessed by liver function tests. However, no study had yet reported finding a dose-response relationship between arsenic and serum hepatic enzymes using different arsenic exposure metrics. To determine risk and the magnitude of the effect of a toxic substance, the assessment of dose-response relationship is critically important. Therefore, the aim of our study was to investigate the dose-response relationship between arsenic exposure and the activities of major serum enzymes used for LFTs in the individuals living in arsenic-endemic areas in Bangladesh.

## Methods

### Study areas and study subjects

Ethical permission for a human study was granted by the Bangladesh Medical Research Council (Mohakhali, Dhaka-1212). Confidentialities and rights of the study subjects were strictly maintained in accord with the guidelines of the Bangladesh Medical Research Council. Arsenic-endemic areas for this study were chosen as described previously [35]. Residents from arsenic-endemic areas included Marua in the Jessore District; Dutpatila, Vultie and Kestopur in the Chuadanga District; and Bheramara in the Kushtia District of the northwest region of Bangladesh were asked to participate in this study. Our group collected hair and nail specimens from study subjects who spontaneously responded to the query. During the sample collection process, we were blinded to arsenic levels in the drinking water, and to those in the hair and nails of the study participants. We collected all blood and other specimens (including water samples) on the same day for each site. The prevalence of typical skin symptoms of arsenicosis (e.g., melanosis on the skin, hyperkeratosis and hard patches on the palms of the hands and soles of the feet) was very high among the local residents of the areas selected for sampling. Local residents (15-60 years of age) were asked to convene at a convenient location in the area, irrespective of the visible presence of skin lesions. We initially selected the study subjects without regard to symptoms. Then subjects who did exhibit symptoms were first identified by a general physician and then the diagnosis was confirmed by a dermatologist. The physician involved in this study carefully examined various parts of the body to confirm the presence of melanosis or hyperkeratosis. Adults who had lived for at least last five years in an arsenic-affected region of Bangladesh were recruited for this study. Individuals who had a previous and recent history of drug addiction, hepatotoxic drugs, malaria, kalazar, chronic alcohol intake or hepatological diseases were excluded from the study, as such factor can influence the hepatic enzyme activity. Pregnant and lactating mothers were also excluded from this study. Of the 216 individuals who were approached, 13 were excluded according to the exclusion criteria [i.e., study candidates, (n = 5) who had resided in the endemic areas for less than five years, pregnant and lactating mothers (n = 6), and hepatological diseases (n = 2)]; thus, a total of 203 subjects were initially recruited (participation rate, 93.98%). Ultimately, we excluded three additional individuals after the collection of blood samples as those individuals were found to be hepatitis B-positive based on our laboratory findings. The subjects who participated in this study gave their written consent. Household visits were carried out in order to interview residents. Personal interviews of the study subjects were conducted by our research team using a standard questionnaire. Information obtained from the interview included the sources of water for drinking and daily household use; water consumption history; socioeconomic status; occupation; eating habits; cigarette smoking; alcohol and tari (locally fermented coconut palm) intake; personal and family history; history of diseases; physiological complications; major diseases; previous physicians' reports; Body Mass Index (BMI); recent history of agricultural or home insecticide or pesticide exposure; and use of air fresheners, aerosols and/or mosquito coils.

### Assessment of arsenic exposure

Study subjects identified the tube well they used as their primary source of drinking water. Water samples from these tube wells were collected in acid-washed containers after the well was pumped for five minutes, as previously described [40]. The total arsenic concentration in the water samples was determined by ICP-MS after the addition of a solution of yttrium (10 ppb in 1.0% nitric acid) to all water samples as an internal standard for ICP-MS analysis. The ion signals for arsenic and yttrium were monitored at m/z values of 75 and 79, respectively. All samples were determined in triplicate, and the average values were used for the data analysis. The detection limit of As^75 ^was 0.03 μg/L. River water (NMIJ CRM 7202-a No.347 National Institute of Advanced Industrial Science and Technology, Japan) was used as a certified reference material (CRM). The average value (mean ± SD) of arsenic in the river water determined in triplicate by ICP-MS analysis, was 1.06 ± 0.04 μg/L (reference value, 1.18 μg/L). In addition, a "cumulative arsenic index" (CAI) [41] was calculated for all known tube wells as (CAI in mg = [arsenic concentration in water of known well, mg/L] × [daily amount of water consumed from that well, L per day] × [duration of well use, days]).

Arsenic levels in the nails and hair have been reported to provide integrated measures of arsenic exposure [35,42,43]. Therefore, for the present study, we assessed hair and nail arsenic concentrations as exposure metrics, together with our assessment of the water arsenic concentrations. Nail samples were collected from each study subject as described previously [44]. Hair samples (length, approximately 1 cm) were collected from a region of the head close to the scalp behind the ear by using a ceramic blade cutter, and the samples were kept in polypropylene bottles [45]. Nail and hair samples were cleaned according to the method described by Chen and colleagues [46]. Samples were immersed in 1% Triton X-100, sonicated for 20 minutes, and then washed five times with milli-Q water. The washed samples were allowed to dry at 60°C overnight in a drying oven. The nail and hair samples were then digested with concentrated nitric acid using a hot plate at 70°C for 15 minutes and at 115°C for 15 minutes. After cooling, the samples were diluted with 1.0% nitric acid containing yttrium (10 ppb), and concentrations of As^75 ^and Y^79 ^in these samples were determined by ICP-MS (HP-4500, Agilent Technologies, Kanagawa, Japan). The accuracy of arsenic measurement was verified using a CRM "cod fish powder" (NMIJ CRM 7402-a, National Institute of Advanced Industrial Science and Technology, Japan). The average value (mean ± SD) of arsenic in the cod fish powder, 34.9 ± 2.35 μg/g (reference value, 36.7 μg/g), was determined in triplicate by the above-mentioned digestion method, followed by ICP-MS analysis.

### Collection of serum and examination for Hepatitis B

All study subjects were asked to convene at a designated location nearby. Fasting blood samples were collected from the study subjects. Blood samples (5-7 ml) were collected from each individual by venipuncture into blood collection tubes. The blood samples were left at room temperature for 30 minutes for clotting and were subsequently centrifuged at 1,200 × g for 20 minutes. The serum supernatant was then taken and stored at - 80°C. All serum samples were checked for hepatitis B using a 3^rd^-generation HBsAg ELISA test kit (Medivent Diagnostic & Co. Ltd., Ireland) according to the manufacturer's protocol.

### Liver function tests of serum hepatic enzyme activity

An analyzer (CHEM-5 V3, Erba, Mannheim, Germany) and commercially available kits were used according to the respective manufacture's protocol for the measurement of serum liver enzyme activity. Serum ALP activity was determined by a kit from BioSystems, SA. Spain. Serum AST and ALT activities were measured by kits from Human, Germany. All samples were analyzed in triplicate, and then mean values were determined.

### Statistical analysis

Statistical analysis for this study was performed using the software Statistical Packages for Social Sciences (SPSS version 17.0, SPSS Inc., Chicago, IL). Descriptive characteristics of the study subjects were collected using several statistical tools. Study subjects were split into quartile groups based on four different concentrations of arsenic in the water, as well as in the hair and nails; for each sampling, the quartile groups were as follows: lowest, low, medium and high. Comparisons of the years of arsenic exposure, and ALP, AST and ALT activities in each group of study subjects were performed by F-test. Median age, water arsenic concentration, cumulative arsenic index and BMI for each group was assessed by the Kruskal Wallis test, and the prevalence of skin symptoms and smoking status were compared by the Chi-square test. The nature of any association between the arsenic exposure metrics and liver enzyme activity was evaluated by scatter plot analysis. Serum ALP, AST and ALT activities for the lowest, low, medium and high quartiles were analyzed by multivariate log linear regression. Log linear regression analyses used in this study were performed before and after adjustments were made for covariates. Normality of the levels of serum hepatic enzyme activity was verified by Q-Q plot. Study subjects were further sub-divided into three groups (≤ 10 μg/L, 10.1-50 μg/L and > 50 μg/L) based on the regulatory upper limit for water arsenic concentrations set by the World Health Organization (WHO, 10 μg/L) and the Bangladesh Government (50 μg/L). Serum hepatic enzyme activities in the three groups were evaluated by one-way ANOVA (Dunnett's T3 test).

## Results

### General characteristics of study subjects

Table 1 shows the characteristics of the study subjects. Study subjects were split into quartile and assigned as groups of lowest, low, medium and high exposure based on arsenic concentrations in the drinking water. The average ages in the lowest, low, medium and high exposure groups were 39.14 ± 11.49, 40.78 ± 12.17, 36.04 ± 10.94 and 37.9 ± 12.5 years, respectively. Most of the male study subjects in the four groups were farmers, whereas most of the female study subjects were housewives. The median (IQR) of water arsenic concentrations in the lowest, low, medium and high exposure groups were 5.3 (3.2-7.29), 93.0 (69.4-117), 186.0 (168-214) and 351.5 (294-527), respectively. There were no significant differences in the average (mean ± SD) BMI of the four groups of study subjects. None of the study subjects had admitted to drinking alcohol on our questionnaire. We found a high percentage of study subjects who had skin symptoms, i.e., 78% for lowest, 94% for low, 60% for medium and 90% for high-exposure group and these results were significantly different (p < 0.01) from those of the respective group of study subjects who had no symptoms. The average years (mean ± SD) of drinking water from the tube wells currently used by the four groups of study subjects were: 16.92 ± 12.03 for lowest, 16.08 ± 9.78 for low, 14.2 ± 8.77 for medium and 13.34 ± 9.17 for high groups, and these differences were not statistically significant. Cumulative arsenic exposure was found to be increased significantly in the higher quartiles.

**Table 1:** General characteristics of the study subjects based on water arsenic levels

Characteristics	Lowest (0.11 - 24.7 μg/L)	Low (34 - 142 μg/L)	Medium (145 - 242 μg/L)	High (249 - 546 μg/L)	*p*-value
No. of subjects	50	50	50	50	

Sex [no. (male/female)]	32/18	31/19	28/22	30/20	

Age [years (Mean ± SD)]	39.14 ± 11.49	40.78 ± 12.17	36.04 ± 10.94	37.9 ± 12.5	0.40**^†^**
Median (IQR^a^)	39 (30 - 47)	35 (30 - 55)	35 (28 - 43)	38 (27 - 45)	

Water As concentration (μg/L) (Mean ± SD)	7.51 ± 7.32	92.89 ± 32.06	191.57 ± 31.03	393.34 ± 107.89	< 0.01**^†^**
Median (IQR^a^)	5.3 (3.2 - 7.29)	93.0 (69.4 - 117)	186.0 (168 - 214)	351.5 (294 - 527)	

Cumulative As index (mg) (Mean ± SD)	193.33 ± 39.82	2361.32 ± 290.31	3751.4 ± 325.13	7466.72 ± 782.04	< 0.01**^†^**
Median (IQR^a^)	58.04 (31.13 - 224.48)	1600.89 (934.91 - 2979.5)	3125.86 (2119.92 - 4777.12)	5515.52 (3007.42-11524.88)	

Years of arsenic exposure (Mean ± SD)	16.92 ± 12.03	16.08 ± 9.78	14.2 ± 8.77	13.34 ± 9.17	0.26*

Occupation					
Female					
Housewives (%)	100	84.21	90.91	85	
Others (%)[Farmworkers, tailors etc.]	-	15.79	9.09	15	
Male					
Farmers (%)	87.5	87.1	92.86	90	
Others (%) [Venders, rickshaw pullers etc.]	12.5	12.9	7.14	10	

(+) symptomps [% (Melanosis and hyperkeratosis)]	78	94	60	90	< 0.01^‡^
(-) symptomps (%)	22	6	40	10	

Smoking habit (%)					
Yes	18	34	8	10	< 0.01^‡^
No	82	66	92	90	

Alcohol intake	-	-	-	-	-

BMI^b ^(Mean ± SD)	21.41 ± 3.54	20.39 ± 3.16	21.23 ± 3.31	20.54 ± 3.26	0.32**^†^**
Median (IQR^a^)	21.45 (18.32 - 21.45)	19.66 (18.04 - 22.24)	20.66 (18.78 - 24.13)	19.96 (18.37 - 21.97)	

ALP activity (U/L) (Mean ± SD)	84.72 ± 22.67	93.99 ± 24.48	100.40 ± 22.19	126.48 ± 35.03	< 0.01*
Median (IQR^a^)	84.17 (68.90 - 96.90)	92.73 (77.87 - 106.03)	97 (85.51 - 113.13)	123.6 (102.7 - 157.48)	

AST activity (U/L) (Mean ± SD)	34.98 ± 7.65	36.19 ± 7.29	37.14 ± 8.20	49.98 ± 25.31	< 0.01*
Median (IQR^a^)	35 (29.14 - 41)	35.58 (30.43 - 40.07)	34.65 (31.05 - 42)	42.5 (36 - 53.5)	

ALT activity (U/L) (Mean ± SD)	24.27 ± 7.69	26.91 ± 10.72	30.82 ± 15.08	43.62 ± 4.90	< 0.01*
Median (IQR^a^)	23.18 (19.22 - 26.84)	24.22 (20.57 - 32.09)	27.93 (21.64 - 36)	32 (25 - 46)	

As = Arsenic; IQR^a ^= Interquartile range; BMI^b ^= Body Mass Index was calculated as body weight (kg) divided by body height squared (m^2^); *p-, ^‡^*p*- and **^†^***p *-values were from the F-test, Chi-square test and Kruskal Wallis test, respectively.

### Correlations of water arsenic concentrations with arsenic concentrations in hair and nails

**Figure 1:**
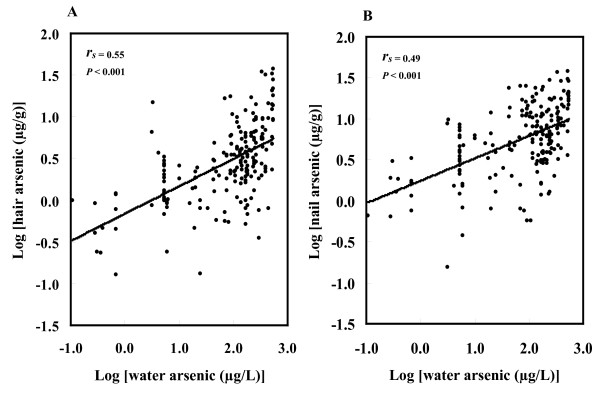
**Relationship between drinking water, hair and nail arsenic concentrations**. Relationship between drinking water arsenic and the arsenic in hair (A) or in nails (B) of the study subjects. Arsenic concentrations in the water, hair and nails were used after log transformation. *r_s _*and *p*- values were from Spearman correlation coefficient test.

The correlation between arsenic in the water with that in hair or nails was based on logarithmic-transformed values. Water arsenic concentrations showed a positive significant relationship (*r*_s _= 0.55, *p *< 0.001) with hair arsenic concentrations (Figure 1A) among the study subjects. Similarly, a positive relationship (*r*_s _= 0.49, *p *< 0.001) was also observed between water and nail arsenic concentrations (Figure 1B).

### Arsenic exposure and serum hepatic enzyme activity

Table 2 shows the exposure-response relationship between water arsenic concentrations and serum enzymes on LFTs, i.e., we evaluated the activities of three liver enzymes in quartile groups of study subjects based on four concentrations of water arsenic. Intriguingly, we found that ALP and ALT activities (Table 2) were significantly increased in the medium- and high-exposure groups before and after adjustments were made for covariates (age, sex, BMI, smoking habit and skin lesions), as compared to lowest (reference) group. In addition, AST activity was found to be significantly increased only in the high-exposure group as compared to the counterpart with the lowest exposure. We then investigated the dose-response relationship between the internal exposure metrics (hair and nail arsenic concentrations) and serum hepatic enzymes. Levels of all three enzymes were significantly higher in the medium- and high-exposure groups than in the lowest group of hair arsenic concentrations (Table 3). In the case of nail arsenic concentrations (Table 4), ALP activity was significantly higher in the medium- and high-exposure groups than in the lowest group. However, significant increases in AST and ALT activities were observed in the high-exposure group compared to the group with the lowest exposure, after adjustments were made for different covariates.

**Table 2:** Comparison of the activities of serum hepatic enzymes in the quartile water arsenic exposure groups by multivariate linear regression analyses

Dependent variables	Independent variable (Water As)	Before adjustment for covariates			After adjustment for covariates^b^		
		Coefficient (95% CI)	*p*-value (t-test)	R^2^	Coefficient (95% CI)	*p*-value (t-test)	R^2^
**ALP**	Lowest (0.11 - 24.7)	-	-	0.230	-	-	0.279
	Low (34 - 142)	0.051 (0.005 - 0.097)	< 0.05		0.039 (- 0.008 - 0.085)	0.11	
	Medium (145 - 242)	0.082 (0.036 - 0.129)	< 0.01		0.087 (0.041 - 0.134)	< 0.01	
	High (249 - 546)	0.175 (0.129 - 0.222)	< 0.01		0.165 (0.119 - 0.212)	< 0.01	

**AST**	Lowest (0.11 - 24.7)	-	-	0.172	-	-	0.217
	Low (34 - 142)	0.016 (-0.028 - 0.061)	0.47		0.025 (- 0.020 - 0.070)	0.27	
	Medium (145 - 242)	0.027 (- 0.018 - 0.071)	0.24		0.023 (- 0.022 - 0.067)	0.32	
	High (249 - 546)	0.13 (0.086 - 0.175)	< 0.01		0.124 (0.080 - 0.169)	< 0.01	

**ALT**	Lowest (0.11 - 24.7)	-	-	0.135	-	-	0.207
	Low (34 - 142)	0.035 (- 0.037 - 0.107)	0.34		0.060 (- 0.011 - 0.132)	0.10	
	Medium (145 - 242)	0.085 (0.013 - 0.157)	< 0.05		0.071 (0.00 - 0.143)	0.05	
	High (249 - 546)	0.189 (0.117 - 0.261)	< 0.01		0.186 (0.115 - 0.257)	< 0.01	

^b^Adjusted for age, sex, BMI, smoking habit and skin lesions; Log-transformed dependent and independent variables were used; Degrees of freedom (df) before and after adjustments were made for covariates, (3, 196) and (8, 191). Units of water arsenic, μg/L; ALP, AST and ALT activities, U/L.

**Table 3:** Comparison of the activities of serum hepatic enzymes in the quartile hair arsenic exposure groups by multivariate linear regression analyses

Dependent variables	Independent variable (Hair As)	Before adjustment for covariates			After adjustment for covariates^b^		
		Coefficient (95% CI)	*p*-value (t-test)	R^2^	Coefficient (95% CI)	*p*-value (t-test)	R^2^
**ALP**	Lowest (0.05 - 1.43)	-	-	0.135	-	-	0.172
	Low (1.57 - 2.80)	0.040 (-0.009 - 0.089)	0.11		0.028 (-0.023 - 0.078)	0.28	
	Medium (2.81 - 5.52)	0.049 (0.000 - 0.098)	0.05		0.044 (-0.005 - 0.093)	0.08	
	High (5.66 - 37.24)	0.134 (0.085 - 0.183)	< 0.01		0.117 (0.066 - 0.168)	< 0.01	

**AST**	Lowest (0.05 - 1.43)	-	-	0.097	-	-	0.150
	Low (1.57 - 2.80)	0.018 (-0.029 - 0.064)	0.46		0.028 (-0.020 - 0.075)	0.25	
	Medium (2.81 - 5.52)	0.054 (0.007 - 0.10)	< 0.05		0.054 (0.008 - 0.099)	< 0.05	
	High (5.66 - 37.24)	0.100 (0.053 - 0.146)	< 0.01		0.096 (0.049 - 0.144)	< 0.01	

**ALT**	Lowest (0.05 - 1.43)	-	-	0.079	-	-	0.165
	Low (1.57 - 2.80)	0.037 (-0.038 - 0.111)	0.33		0.064 (-0.010 - 0.139)	0.09	
	Medium (2.81 - 5.52)	0.084 (0.010 - 0.159)	< 0.05		0.086 (0.014 - 0.158)	< 0.05	
	High (5.66 - 37.24)	0.146 (0.071 - 0.220)	< 0.01		0.151 (0.076 - 0.225)	< 0.01	

^b^Adjusted for age, sex, BMI, smoking habit and skin lesions; Log-transformed dependent and independent variables were used; Degrees of freedom (df) before and after adjustments were made for covariates, (3, 196) and (8, 191). Units of hair arsenic, μg/g; ALP, AST and ALT activities, U/L.

**Table 4:** Comparison of the activities of serum hepatic enzymes in the quartile nail arsenic exposure groups by multivariate linear regression analyses

Dependent variables	Independent variable (Nail As)	Before adjustment for covariates			After adjustment for covariates^b^		
		Coefficient (95% CI)	*p*-value (t-test)	R^2^	Coefficient (95% CI)	*p*-value (t-test)	R^2^
**ALP**	Lowest (0.15 - 3.17)	-	-	0.120	-	-	0.146
	Low (3.21 - 6.25)	0.043 (-0.007 - 0.092)	0.09		0.037 (-0.014 - 0.088)	0.15	
	Medium (6.26 - 11.27)	0.071 (0.021 - 0.121)	< 0.01		0.057 (0.005 - 0.110)	< 0.05	
	High (11.4 - 37.42)	0.127 (0.077 - 0.177)	< 0.01		0.109 (0.055 - 0.163)	< 0.01	

**AST**	Lowest (0.15 - 3.17)	-	-	0.049	-	-	0.116
	Low (3.21 - 6.25)	- 0.014 (-0.062 - 0.034)	0.57		- 0.007 (-0.055 - 0.040)	0.77	
	Medium (6.26 - 11.27)	0.005 (-0.042 - 0.053)	0.83		0.010 (-0.039 - 0.060)	0.68	
	High (11.4 - 37.42)	0.058 (0.010 - 0.105)	< 0.05		0.062 (0.012 - 0.113)	< 0.05	

**ALT**	Lowest (0.15 - 3.17)	-	-	0.012	-	-	0.113
	Low (3.21 - 6.25)	0.027 (-0.050 - 0.104)	0.50		0.043 (-0.033 - 0.118)	0.27	
	Medium (6.26 - 11.27)	0.029 (-0.048 - 0.106)	0.46		0.055 (-0.024 - 0.133)	0.17	
	High (11.4 - 37.42)	0.061 (-0.016 - 0.138)	0.12		0.084 (0.004 - 0.165)	< 0.05	

^b^Adjusted for age, sex, BMI, smoking habit and skin lesions; Log-transformed dependent and independent variables were used; Degrees of freedom (df) before and after adjustments were made for covariates, (3, 196) and (8, 191). Units of nail arsenic, μg/g; ALP, AST and ALT activities, U/L.

### Serum hepatic enzyme activities based on the regulatory upper limit of arsenic concentrations in drinking water

We then divided our study population into three groups (≤ 10 μg/L, 10.1-50 μg/L and > 50 μg/L) based on the regulatory upper limit of water arsenic concentrations set by the WHO (10 μg/L) and the Bangladesh Government (50 μg/L) in order to evaluate the serum hepatic enzymes in these three groups (Table 5). We found that the levels of three enzymes tested in this study were significantly higher in the > 50 μg/L group than in the two other groups. Interestingly, increased levels of serum hepatic enzyme activities were observed in the 10.1-50 μg/L group compared to the ≤ 10 μg/L group, although these differences were not statistically significant.

**Table 5:** Serum hepatic enzyme activities in the three groups based on the regulatory upper limit of arsenic concentrations in drinking water

Parameters	Categories	n	Liver Enzyme Activity (U/L) (Mean ± SD)		
			**ALP**	**AST**	**ALT**

Arsenic in drinking water	≤ 10 μg/L	38	83.37 ± 24.27	34.67 ± 7.42	24.23 ± 7.91
	
	10.1 - 50 μg/L	21	90.47 ± 18.14	35.86 ± 8.00	26.92 ± 8.41
	
	> 50 μg/L	141	107.75 ± 31.48^a, b^	41.45 ± 17.42 ^a, b^	34.01 ± 24.26 ^a, b^

*p*-value			< 0.01^a^, < 0.01^b^	< 0.01^a^, 0.05^b^	< 0.01^a^, < 0.05^b^

^a^Statistically significant ≤ 10 μg/L

^b^Statistically significant from between 10.1 and 50 μg/L

## Discussion

The ingestion of inorganic arsenic via drinking water is known to be associated with liver damage, liver cancer and other types of liver dysfunction [36,47-49]. Hepatic disorders appear to be one of the major causes of arsenic-related mortality [27-29]. Although previous epidemiologic studies have demonstrated liver toxicity by arsenic exposure [37-39], serum enzyme activity as recognized on LFTs showing a dose-response relationship has not yet been clearly documented. The arsenic-endemic northwest region of Bangladesh was chosen for the selection of subjects for this study. Here, we found that hair and nail arsenic levels were positively correlated with arsenic levels in drinking water (Figure 1), and these correlations were statistically significant. These results are in agreement with those of previous reports [35,42-44,50], which suggests that arsenic contents in hair and nail samples might be used as effective biomarkers of arsenic exposure.

We found a wide range of arsenic concentrations in the drinking water of the study subjects (0.11-546 μg/L). This wide range of water arsenic concentrations led us to evaluate the dose-response relationship between arsenic exposure and serum hepatic enzyme activity. The study subjects were separated into quartile groups based on four different concentrations of arsenic in the drinking water. We observed that ALP activity significantly increased with each increasing arsenic-exposure group (Table 2); however, the other two enzymes (AST and ALT) assessed were only significantly increased in the high-exposure groups. Serum ALP is not specific to liver injury alone. However, when elevated serum ALP is found together with elevated serum AST and ALT levels, which are more specific to liver damage, the results could be indicative of liver damage. Since the relationship between water arsenic and serum hepatic enzymes suggested only an external exposure-response relationship, we next examined the dose-response relationship using candidate biomarkers (hair and nails) of arsenic exposure. Similar patterns of ALP activity were seen with increasing levels of hair and nail arsenic concentrations, before and after adjusting for different covariates (Tables 3 and 4). In the case of AST and ALT levels, there were some small differences in terms of the shape of the dose-response relationship in the hair and nail arsenic categories. After adjustments were made for covariates, AST and ALT activities were significantly higher in the medium- and high-exposure groups than in the group with the lowest level of exposure according to the arsenic concentrations in the hair samples. Moreover, it was established that the activities of the two enzymes (AST and ALT) were significantly increased only in the high-exposure group compared to the lowest-exposure group according to the arsenic concentration in the nail samples. These small differences in dose-response relationship between serum hepatic enzyme activities and hair and nail arsenic concentrations were probably due to the temporality of the arsenic exposure metrics. Hair concentrations represent immediate exposure, as one centimeter of hair reflects approximately one month of exposure. On the other hand, nails capture historical exposure from several months to a year prior to sampling, there remains the possibility that exposure of the study subjects was inconsistent for some period during the past year. All of the results clearly demonstrated that arsenic exposure was associated with liver toxicity, as indicated by increased serum liver enzyme activity levels. The results of the present study were consistent with the results of other studies of toxic exposure to heavy metals such as lead [51,52]. The major strengths of the present study were to demonstrate the particular effects of arsenic on serum hepatic enzymes by consideration of three different exposure metrics (water, hair and nail arsenic levels).

There are several factors, including alcohol intake, age, sex, BMI and smoking habit that may influence hepatic enzyme activity [53-56]. This study excluded the probability of the confounding effect of alcohol consumption on serum hepatic enzymes, as no participants in this study drank alcohol (Table 1), primarily due to the social and religious restrictions on alcohol intake in Bangladesh. For all associations, adjustments were made for covariates (age, sex, BMI, smoking habit and skin lesions), and the results explicitly demonstrated that arsenic exposure was the main contributor to the increasing serum levels of hepatic enzyme activity. Although this study represents extensive epidemiological research efforts to determine the effects of arsenic exposure on serum hepatic enzymes, there were some potential limitations warranting further discussion. First, there might have been certain inaccuracies in calculating the cumulative arsenic index due to erroneous recall by study participants of their drinking water history. However, we saw related associations with hair and nail arsenic concentrations, both of which are candidate biomarkers of exposure. Second, we selected study subjects to exclude those who had a past or present history of hepatitis, hepatotoxic drugs, or jaundice. Moreover, three individuals were excluded from the analysis after laboratory examination for hepatitis B had been conducted. The general practitioner involved in this study carefully determined whether the study subjects were suffering from jaundice or other types of liver disease; however, we did not perform laboratory examinations for additional confirmation of jaundice or other forms of hepatitis. Third, it has been reported that nutritional status can influence the effects of arsenic on health [37-39,42,43,57,58]. In this study, nutritional status was not taken into account as a covariate, as the study subjects were selected from the same socioeconomic group, and the participants were similar to each other in terms of food intake (data not shown). It should be noted, however, that individual differences in nutritional status could have influenced the results observed in this study. Further studies will be needed to confirm the effects of individual nutritional status and its association with liver enzyme levels. Fourth, we did not assess the confounding effects of other metals present in the water on the observed association between arsenic exposure and serum hepatic enzymes. All study subjects (i.e., lowest, low, medium and high exposure) were from the same arsenic-endemic areas. If any other metals present in the drinking water could have affected the association between arsenic exposure and liver enzyme activity, concentrations of those other metals would have paralleled the same concentration gradients as those of arsenic in the drinking water, hair and nails. This is unlikely, however; more detailed investigations into the effects of other metals will be needed in the future. Fifth, study participants who lived in arsenic-endemic areas for at least five years and who had a high prevalence of skin lesions (80.5%) were selected for this study. Therefore, the findings of the current study may be generalizable to other studies, provided that the study samples adhered to the same criteria.

Environmental exposure to arsenic is unavoidable and medicinal use of arsenicals in the treatment of certain cancers is increasing. The International Agency for Research on Cancer listed the liver as a potential organ for arsenic carcinogenesis. Arsenic-induced liver dysfunction, hepatomegaly and liver fibrosis warrant increased attention, as these preneoplastic changes could advance to malignancy. The serum hepatic enzyme levels assessed in conventional LFTs may be appropriate tools to achieve a prognosis for arsenic-induced, early preneoplastic changes in the liver. Early prognosis of liver dysfunction may reduce the incidence of arsenic-related liver cancer, as well as the related mortality. Furthermore, in all such cases of toxicity, knowing the dose-response relationship is a necessary part of understanding the cause-and-effect relationship between chemical exposure and illness. While there have been several studies showing that arsenic exposure affects liver function, good dose-response data are not available. The present study was conducted in the population of arsenic-endemic areas in Bangladesh, where there is a wide range of arsenic levels in the drinking water (range, 0.11 - 546 μg/L). To the best of our knowledge, this is the first systematic study to evaluate the dose-response relationship between arsenic exposure and serum enzymes for LFTs by considering three different exposure metrics, i.e., water, hair and nail arsenic concentrations in individuals living in arsenic-endemic areas in Bangladesh. The water arsenic threshold set by the Bangladesh Government is five times higher (50 μg/L) than suggested in the WHO standard guidelines (10 μg/L). Therefore, in this study, hepatic enzyme activities among three groups of individuals whose drinking water contained arsenic levels ≤ 10 μg/L, 10.1-50 μg/L and > 50 μg/L were assessed. The activities of the three enzymes tested in this study (ALP, AST and ALT) were significantly higher in the > 50 μg/L group than in the two other groups (Table 5). Interestingly, elevated levels of hepatic enzyme activity were observed in the 10.1 - 50 μg/L exposure group, as compared to the ≤ 10 μg/L exposure group. Although the differences between these two groups were not statistically significant, these results might still be of note from a policy perspective.

## Conclusions

The present study demonstrated that water arsenic concentrations were strongly correlated with hair and nail arsenic concentrations. Serum hepatic enzymes (ALP, AST and ALT) used for the LFTs were elevated in the higher exposure groups as compared to the group with the lowest exposure to arsenic in the drinking water. Similarly, levels of serum hepatic enzyme activity were also increased in the higher exposure groups of arsenic in the hair and nails. Thus, the results of this study provided new insights into arsenic-induced liver toxicity. These findings might be helpful for the early detection of arsenic exposure-related liver diseases in people living in arsenic-endemic regions.

## Abbreviations

LFTs: Liver Function Tests; ICP-MS: Inductively Coupled Plasma Mass Spectroscopy; ALP: alkaline phosphatase; AST: aspartate transaminase; ALT: alanine transaminase; WHO: World Health Organization

## Competing interests

The authors declare that they have no competing interests.

## Authors' contributions

KI and AH were involved in the planning of the study, data management, laboratory experiment and manuscript preparation. MRK, PS and NA were participated in the discussion of the methodology, statistical analyses and interpretation of the results. AF, EH, KAS and MR assisted in acquisition of data and carried out laboratory experiment. ZAS, MR, MRK and AAA contributed to collecting the specimens, to defining the objectives of the analysis and to revising the manuscript. MH, as a clinical doctor, was involved in conception and design of the study, medical data collection, characterization of the study subjects. AM was involved in the drafting and critical review of the manuscript. HM and SH were involved in the exposure assessment and in the design of the study. KH took the overall responsibility in hypothesis generation, study design, data analysis and manuscript preparation. All authors read and approved the final manuscript.
